# Von Willebrand factor and *JAK2*V617F variant allele frequency predict thrombosis and bleeding in patients with myeloproliferative neoplasms

**DOI:** 10.1007/s00277-025-06648-9

**Published:** 2025-10-14

**Authors:** Ayala Gat, Romi Kazoom, Inbal Shapira, Ela Shai, Batia Roth Jelinek, Noa Buchman, Yosef Kalish, Ariela Arad, Eran Zimran

**Affiliations:** 1 Faculty of Medicine, Hebrew University of Jerusalem, Jerusalem, Israel; 2 Department of Hematology, Hadassah Medical Center, Faculty of Medicine, Hebrew University of Jerusalem, Jerusalem, Israel

**Keywords:** Myeloproliferative neoplasms, Von willebrand factor, *JAK2*V617F variant allele frequency, Thrombosis, Bleeding

## Abstract

Thrombosis is a major cause of morbidity and mortality in patients with myeloproliferative neoplasms (MPN), while bleeding is also common. Current thrombosis and bleeding risk stratification strategies are limited, creating a need for identification of novel risk factors to better predict these complications. We performed laboratory coagulation testing and driver mutation profiling from peripheral blood of patients with MPN. Patients’ history of comorbidities, hematological variables and thrombosis and/or bleeding events were collected retrospectively at the time of testing. Patients were then followed for a median of 3.3 years for incident events. 198 patients were included in the study. Thrombotic events occurred in 41% of the patients. Age, cardiovascular comorbidities and constitutional symptoms were significantly associated with thrombosis, as well as elevated von Willebrand Factor (vWF): Antigen, vWF: Activity and factor VIII levels. Elevated vWF: Antigen levels remained independent in multivariate analysis and were predictive of incident thrombosis. Bleeding events occurred in 26% of the patients. vWF: Activity ≤ 30%, vWF: Activity-to-antigen ratio of ≤ 0.7, a platelet count of ≥ 500*10^9^/L and *JAK2*V617F variant allele frequency (VAF) of ≥ 50% were predictive of total (including minor) bleeding events. However, only *JAK2*V617F VAF of ≥ 50% was predictive of major and clinically relevant non-major bleeding. Extreme thrombocytosis defined by a platelet count of ≥ 1000*10^9^/L was not associated with bleeding or acquired vWF syndrome. vWF profiling and *JAK2*V617F VAF contribute to thrombosis and bleeding risk assessment in patients with MPNs and should be incorporated routinely at diagnosis and during follow-up.

## Introduction

Arterial and venous thrombosis are a major cause of morbidity and mortality in the Philadelphia-negative myeloproliferative neoplasms (MPN) including polycythemia vera (PV), essential thrombocythemia (ET) and primary or post-PV or -ET myelofibrosis (collectively, MF) [1]. MPN-related thrombosis is characterized by a high prevalence of thrombotic events preceding diagnosis, a persistent risk of thrombosis following diagnosis remaining high even under optimal treatment, and an extremely strong association with atypical-site venous thrombosis [2–5]. Prevention of thrombosis is a crucial aspect of management; however, current assessment scores rely on simple clinical criteria and are extremely limited [6].

The most well-established risk factors for thrombosis in MPNs are age over 60 and history of previous thrombosis in both PV and ET, as well as elevated hematocrit in PV and presence of the *JAK2*V617F mutation in ET [6]. Other relevant risk factors include cardiovascular comorbidities, splenomegaly, a high need of phlebotomies, leukocytosis and neutrophil-to-lymphocyte ratio [7–12]. Molecular findings such as *JAK2*V617F variant allele frequency (VAF) and additional somatic mutations in myeloid-related genes are under active investigation [13–15]. Laboratory markers of coagulation activation and thromboinflammation have been proposed as potential predictors of thrombotic risk; but have not been extensively studied [16].

Von Willebrand factor (vWF) is a large multimeric glycoprotein that has a dual role in hemostasis: it promotes adhesion of platelets by acting as a ligand for their glycoproteins and serves as the carrier and protector for coagulation factor VIII (FVIII). Both vWF and FVIII are overexpressed in inflammatory states [17]. Elevated levels of vWF have been associated with venous as well as arterial thrombotic events in non-MPN patients but little is known about its relevance in MPN [18, 19].

Despite the high risk of thrombosis, some patients with MPN paradoxically suffer from a bleeding tendency, further complicating management. Bleeding in MPN has historically been associated with an acquired deficiency of vWF linked to high platelet counts, however, other mechanisms may exist [20]. We previously observed a high rate of bleeding in a series of patients with PV and ET, associated with the acquired von-Willebrand syndrome (AVWS). AVWS was not limited to extreme thrombocytosis in either PV or ET and was significantly associated with the presence of *JAK2*V617F in patients with ET. We suggested that vWF testing should be included in the routine evaluation of all patients with MPN regardless of platelet counts [21]. To test this suggestion in the context of both thrombosis and bleeding risk, we performed a laboratory coagulation profile including vWF testing in an unselected and heterogeneous MPN patient population. We hypothesized that coagulation laboratory testing and driver mutation status could be used in the assessment of thrombosis and bleeding risk in patients with MPN.

## Methods

### Study design and patient population

We conducted a single-center observational study of patients aged 18 years or older referred to our center between 2019 and 2024 and enrolled in the Hadassah Medical Organization MPN Database. Patients had a diagnosis of ET, PV or primary or post-PV/ET MF according to the 2016 WHO classification [22]. Peripheral blood samples were collected from study participants attending the hematology clinics or day care at our center either at diagnosis or during periodic evaluation visits over the course of their disease. Patients with cytopenic MF or leukemic transformation, as well as patients with active thrombosis or bleeding at the time of testing, were excluded from the study. The study protocol was approved by the local institutional review board (0533-19-HMO) and all patients provided written informed consent.

## Laboratory testing

Laboratory testing included complete blood counts, biochemistry studies, molecular testing for driver mutation status and coagulation profile. The coagulation profile included prothrombin time (PT/INR), partial thromboplastin time (PTT), fibrinogen, D-dimer and vWF antigen (vWF: Ag) and activity (vWF: Ac) levels, the latter measured by ristocetin cofactor assay. *JAK2*V617F VAF was available in a subset of patients who had undergone NGS myeloid panel testing. Data on patients’ medical history was collected retrospectively from electronic medical records at the time of laboratory testing. Patients were then followed prospectively for incident thrombosis and/or bleeding events and disease progression.

## Thrombosis and bleeding events

Thrombotic events were defined based on ICD-10 codes. Arterial thrombotic events (ATE) included acute coronary syndrome (ACS), ischemic stroke, transient ischemic attack (TIA) and peripheral vascular occlusion. Venous thrombotic events (VTE) included deep vein thrombosis (DVT), pulmonary embolism (PE) and venous thrombosis of atypical locations. Major and clinically relevant non-major bleeding (CRNMB) were defined according to the ISTH criteria [23]. Major bleeding included fatal bleeds or those requiring ≥ 2 RBC units, ≥ 2 g/dL hemoglobin drop, or bleeding at critical sites. CRNMB was defined as non-major bleeding that warranted medical intervention, hospitalization, or a change in therapy. Prevalent and incident events were ascertained with respect to the time of coagulation laboratory testing. Prevalent events were those occurring before laboratory testing, while incident events were defined as events that occurred after laboratory testing, during prospective follow-up. All thrombosis and bleeding events were reviewed by the authors for accuracy.

### Statistical analysis

The association between two categorical variables was tested by using either the Chi-square or the Fisher’s exact tests. Comparing quantitative variables between two and three independent groups was performed by applying the two-sample t-test or the non-parametric Mann-Whitney test, and the non-parametric Kruskal-Wallis test, respectively. The strength of the association between two quantitative variables was assessed by calculating the Pearson as well as the Spearman non-parametric correlation coefficients. The multivariate logistic regression model was applied for simultaneously assessing the effect of several independent variables on a dichotomous dependent variable. To test the effect of categorical variables on dichotomous events, Kaplan-Meier survival analysis was performed with the log-rank test for the comparison of survival curves. All statistical tests applied were two-tailed, and a p-value of 0.05 or less was considered statistically significant. Data was analyzed using R version 4.4.3.

## Results

### Patient characteristics

This study included 198 patients; baseline characteristics are summarized in Table 1. The mean age at the time of laboratory testing was 62 years and 51% were female. The mean time from MPN diagnosis to laboratory testing was 9.8 years and the mean follow-up after testing was 3.3 years. MPN disease entities included PV (44%), ET (29%) and primary or post-ET or -PV myelofibrosis (27%). 81% of the patients harbored *JAK2*V617F, 11% and 2% harbored *CALR* and *MPL* mutations, respectively, and 6% were negative to all three MPN driver mutations. 74% had at least one comorbidity, the most common being hypertension (43%) and dyslipidemia (32%). Common clinical manifestations included fatigue (68%), splenomegaly (51%) and pruritus (23%). 74% of the patients had received any type of cytoreductive therapy, and 90% were under either anticoagulation and/or antiplatelet agents.

**Table 1:** Patient characteristics

[n, (%) shown as proportions of total study population]	
N=198	
Age at diagnosis (Mean ± SD)	52.6 ± 18.6
Age at time of laboratory testing (Mean ± SD)	62 ± 18.2
Female sex	101 (51%)
Cardiovascular risk factors:	
Hypertension (HTN)	86 (43%)
Diabetes mellitus (DM)	44 (22%)
Dyslipidemia	65 (33%)
Smoking	34 (17%)
Obesity	32 (16%)
≥3 Risk factors	38 (19%)
MPN type:	
Polycythemia vera (PV)	87 (44%)
High risk PV	55 (28%)
Essential thrombocytosis (ET)	57 (29%)
High risk ET	20 (10%)
Myelofibrosis	
Primary myelofibrosis	12 (7%)
Post- PV myelofibrosis	25 (11%)
Post- ET myelofibrosis	17 (9%)
Driver mutation status	
JAK2V617F	160 (81%)
Calreticulin (CALR)	22 (11%)
Myeloproliferative leukemia (MPL)	5 (2.5%)
Triple-negative	11 (5.5%)
MPN signs and symptoms	
Fatigue	134 (68%)
Constitutional symptoms	59 (30%)
Microvascular symptoms	56 (28%)
Pruritus	46 (23%)
Splenomegaly	100 (51%)
Erythromelalgia	7 (3.5%)
Gout	12 (6%)
Treatments	
Anti-platelet agents	153 (77%)
Anticoagulation	49 (25%)
Concomitant anti-platelets and anticoagulation	24 (12%)
No treatment with anti-platelet or anticoagulation	20 (10%)
Phlebotomy	79 (40%)
Hydroxyurea	119 (60%)
Interferons	36 (18%)
JAK Inhibitors	37 (19%)

The distribution and timing of thrombotic and bleeding events are detailed in Table 2. Thrombotic events (TE) at any time occurred in 41% of the patients, comprising of ATEs in 30% and VTEs in 18%. Fourteen patients (7%) had experienced both arterial and venous events. Among ATEs, the most prevalent were ACS in 35%, followed by ischemic strokes, TIAs and splenic infarcts each occurring in 18% of the patients. Among VTEs, 53% were splanchnic vein thrombosis, 42% were DVT and 5% were PE. 37% of the events occurred prior to MPN diagnosis and 53% occurred following diagnosis. Bleeding events at any time occurred in 26% of the patients; 14% had minor bleeding, 6% had major bleeding and 6% had CRNMB.

**Table 2:** Details of thrombosis and bleeding events prior to or following laboratory testing

**Thrombotic event**	**Prior to testing**	**Following testing**	**Total number of events (% of study population)**
Total thrombotic events	68	14	82 (41.4%)
Arterial events	49	11	60 (30.3%)
ACS	19	2	21 (10.6%)
Ischemic stroke	8	3	11 (5.6%)
TIA	9	2	11 (5.6%)
Splenic infarcts	8	3	11 (5.6%)
PVD	5	1	6 (3%)
Venous events	33	3	36 (18.2%)
SVT	17	2	19 (10.1%)
DVT	14	1	15 (7.9%)
PE	2	0	2 (1.1%)
Venous + arterial events			14 (7.1%)
**Bleeding event**	**Prior to testing**	**Following testing**	**Total number of events (% of study population)**
Total bleeding events	29	23	52 (26.3%)
Minor bleeding	17	14	31 (17.7%)
CRNMB	6	5	11 (5.6%)
Major bleeding	6	4	10 (5.6%)

*ACS* acute coronary syndrome, *TIA* transient ischemic attack, *PVD* peripheral vascular disease, *SVT* splanchnic vein thrombosis, *DVT* deep vein thrombosis, *PE* pulmonary embolism, *CRNMB* clinically relevant non major bleeding

*12 patients experienced both major/CRNMB and thrombotic events

## Factors associated with thrombosis

We first analyzed retrospectively the associations between various factors and overall TEs including both prevalent and incident events (Table 3). In univariate analysis, patient age (*p* = 0.006) as well as history of hypertension (OR 2.43, 95% CI 1.36–4.34), diabetes mellitus (OR 1.99, 95% CI 1.01–3.91) and dyslipidemia (OR 2.1, 95% CI 1.17–3.9) were significantly associated with TEs, while sex, history of smoking, obesity and MPN type were not. Constitutional symptoms were also significantly associated with TEs (OR 3.13, 95% CI 1.66–5.89), while microvascular symptoms and splenomegaly were not. Patients harboring *JAK2*V617F exhibited a trend for an association with TEs as compared to non-*JAK2* mutated patients (OR 1.99, 95% CI 0.89–4.42, *p* = 0.086). Although not associated with overall TEs, the *JAK2*V617F VAF was especially high in the small group of patients who experienced both venous and arterial TEs (*p* = 0.026).

**Table 3:** Association of patient variables with overall (prevalent and incident) arterial and venous thrombotic events

Variable	Thrombotic events		
	Yes (n=82)	No (n=116)	p value (univariate)
Female sex (n=101)	46	55	0.12
Age (mean)	67	60	0.006
Comorbidities			
Hypertension (n=86) *	46	40	0.003
Diabetes mellitus (n=44)	24	20	0.045
Dyslipidemia (n=65)	35	30	0.013
Smoking (n=34)	17	17	0.26
Obesity (n=32)	17	15	0.16
MPN type, categorized			
Polycythemia vera (n=87)	35	52	
Essential thrombocytosis (n=57)	21	36	
Myelofibrosis (n=54)	26	28	
MPN manifestations			
Constitutional symptoms (n=59)	36	23	<0.001
Microvascular symptoms (n=56)	23	33	0.92
Spleen enlargement (n=100)	45	55	0.14
Laboratory variables (mean)			
INR (n= 144)**	1.16	1.08	0.06
PTT (sec) (n=144)**	31	30	0.53
D-dimer (mg/L)	0.34	0.27	0.47
Fibrinogen (mg/dL)	313	282	0.15
vWF:Ag (%) *	145	97	<0.001
vWF:Ac (%)	117	71	<0.001
vWF:Ac/Ag	0.68	0.49	0.27
Factor VIII	205	149	<0.001
JAK2V617F positive (n=160)	71	89	0.086
*JAK2*V617F variant allele frequency (% mean) (n=82)	45	42	0.62

*In multivariate analysis, only hypertension and elevated vWF:Ag levels remained significantly associated with thrombosis and were independent of all other factors including age.

** Calculation of PT/INR and PTT excluded patients under anticoagulation

**Fig. 1:**
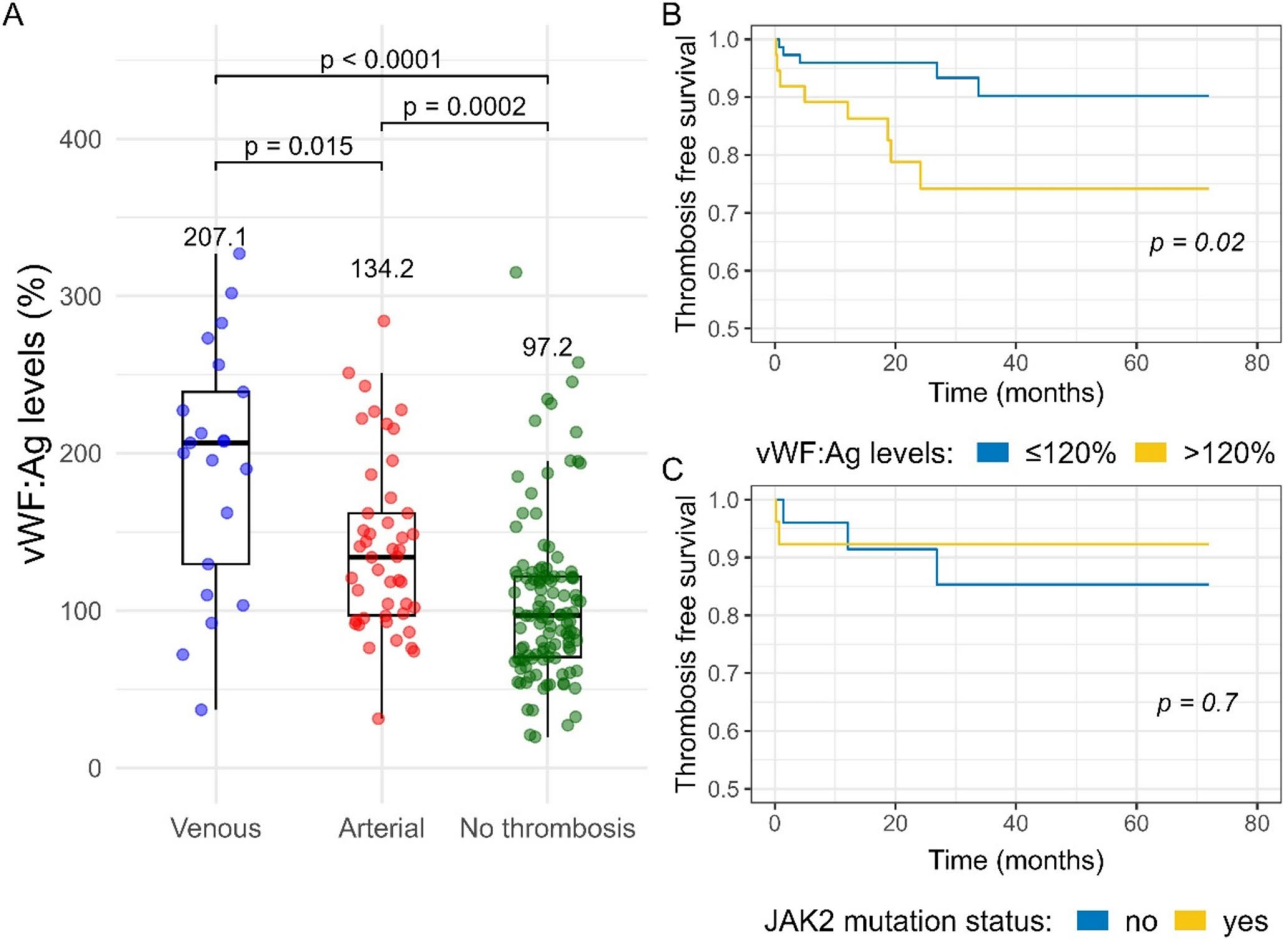
vWF: Ag distribution by type of thrombotic event; (**B**, **C**) Kaplan-Meier analysis of selected risk factors and their association with thrombosis-free survival

Multiple laboratory parameters were assessed for association with TEs (Table 3). The average hemoglobin levels and platelet (PLT) counts were lower in patients who experienced TEs, as compared to those who did not (not shown), an association likely confounded by active cytoreductive therapy. We did not observe an association between WBC counts, CRP, ferritin, D-dimer or fibrinogen levels and thrombosis; however, a strong association was observed between elevated levels of vWF: Ag, vWF: Ac and FVIII and TEs (*p* < 0.001 for all). In multivariate logistic regression analysis that included age along with all the above clinical and laboratory factors, only hypertension and elevated vWF: Ag levels remained independently associated with TEs. Remarkably, elevated vWF: Ag levels were significantly associated with both venous and arterial events, even when analyzed separately; patients with VTEs showed higher vWF: Ag levels as compared to patients with ATEs, which in turn had higher levels as compared to patients without thrombosis (Fig. 1A).

## Analysis of incident thrombosis

To prospectively evaluate the potential use of the above risk factors as predictive markers for thrombosis, we performed Kaplan Meier analyses on 130 patients without previous thrombosis at the time of laboratory testing, using dichotomous variables. Our analysis revealed a significantly inferior thrombosis-free survival in patients with elevated vWF: Ag levels of > 120% (*p* = 0.02, Fig. 1B). Other variables including WBC and PLT counts, *JAK2*V617F status, and abnormal CRP, fibrinogen or D-dimer levels were not associated with thrombosis-free survival (Fig. 1C).

### Factors associated with bleeding

We then investigated the associations between various factors and overall bleeding including both prevalent and incident events (Table 4); this retrospective analysis included minor, major and clinically relevant non-major bleeding events (referred to as ‘total bleeding’). In univariate analysis, patients who experienced bleeding events were younger at diagnosis (*p* = 0.008). Female sex, constitutional symptoms, splenomegaly, and higher *JAK2*V617F VAF were positively associated with total bleeding events. A correlation was also observed between the type of MPN and the occurrence of bleeding; patients with ET or MF had a significantly higher prevalence of bleeding as compared with PV (*p* = 0.024). Conversely, treatment with anticoagulation was not associated with total bleeding events.

**Table 4:** Association of patient variables with overall (prevalent and incident) total bleeding events (minor, clinically relevant non major and major bleeding)

Variable	Bleeding events		
	Yes (n=52)	No (n=146)	p value (univariate)
Female sex (n=101) *	35	67	0.004
Age at diagnosis (mean) *	46	54.3	0.008
Comorbidities			
Hypertension (n=86)	22	64	0.85
Diabetes mellitus (n=44)	10	34	0.55
Dyslipidemia (n=65)	14	51	0.29
Obesity (n=32)**	11	21	0.23
MPN type, categorized			
Polycythemia vera (n=87)	16	71	0.024
Essential thrombocytosis (n=57)	15	42	0.99
Myelofibrosis (n=54)	21	33	0.022
MPN manifestations			
Constitutional symptoms (n=59)	22	37	0.027
Microvascular symptoms (n=56)	17	39	0.37
Spleen enlargement (n=100)	34	66	0.023
Laboratory variables (mean)			
PLT (10^9^/L)	640	525	0.09
PLT>1000 (10^9^/L)	14	24	0.15
vWF:Ag (%)	128	128	0.97
vWF:Ac (%)	86	102	0.11
vWF:Ac/Ag < 0.7	33	53	<0.001
vWF:Ac/Ag < 0.5	16	18	0.004
Factor VIII (IU/dL)	177	172	0.71
*JAK2*V617F positive (n=160)	42	117	0.62
*JAK2*V617F variant allele frequency (% mean) (n=82) *, **	57.5	36.4	0.004
Treatment with anticoagulation	16	33	0.24

*In multivariate analysis: only female sex, age and *JAK2*V617F VAF remained significantly associated with total bleeding events.

**Obesity and *JAK2*V617F VAF were the only variables significantly associated with major and CRNMB (when excluding minor bleeding)

In coagulation laboratory testing, both AVWS defining variables, vWF: Ac levels of ≤ 30% and vWF: Activity-to-antigen ratio (vWF: Ac/Ag) of ≤ 0.7, were significantly associated with a higher prevalence of bleeding (OR 4.3, 95% CI 1.20–9.16, and OR 3.35, 95% CI 1.63–7.07, respectively), while the platelet count was not. In multivariate logistic regression analysis, age at diagnosis, female sex and higher *JAK2*V617F VAF remained significantly associated with total bleeding events and were independent of all other exposures including treatment with anticoagulation.

When restricting the analysis to major and CRNMB events, an association with AVWS was not observed. Only obesity (OR 3.32, 95% CI 1.20–9.16) and higher *JAK2*V617F-VAF were significantly associated with major bleeding and CRNMB. This association was independent of age, platelet counts, vWF: Ac and treatment with anticoagulation. Moreover, among the ten patients who experienced major bleeding events, only two were under active treatment with anticoagulation and one filled criteria for AVWS.

**Fig. 2:**
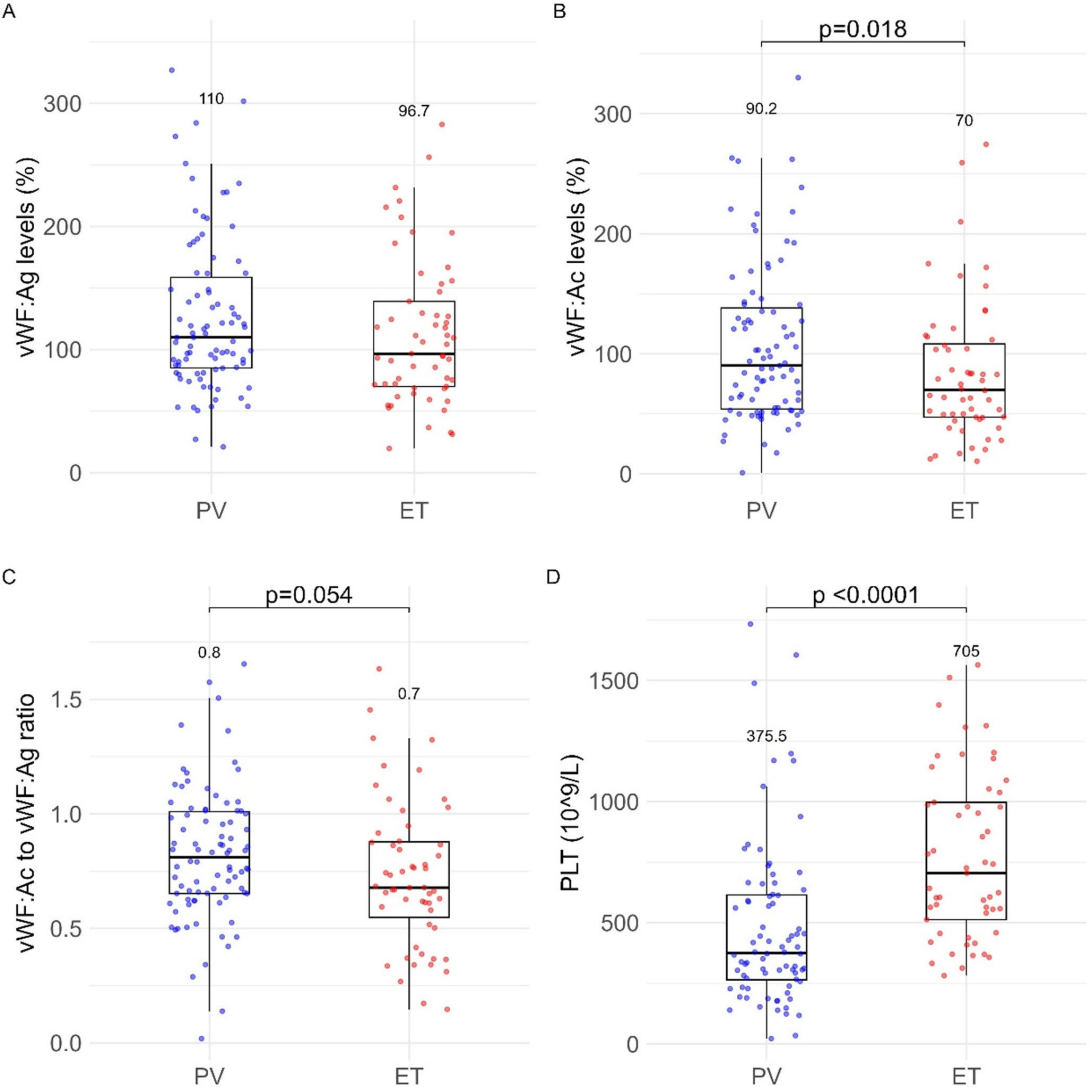
VWF variables and MPN type. (**A**) vWF: Ag levels; (**B**) vWF: Ac levels; (**C**) vWF: Ac to vWF: Ag ratio; (**D**) platelet count

Of note, we observed differences in the vWF profile between MPN types. While patients with PV had higher vWF: Ag levels, patients with ET had significantly lower vWF: Ac and vWF: Ac/Ag, indicating a higher rate of AVWS, that correlated well with a higher mean platelet count (Fig. 2).

### Analysis of incident bleeding

**Fig. 3:**
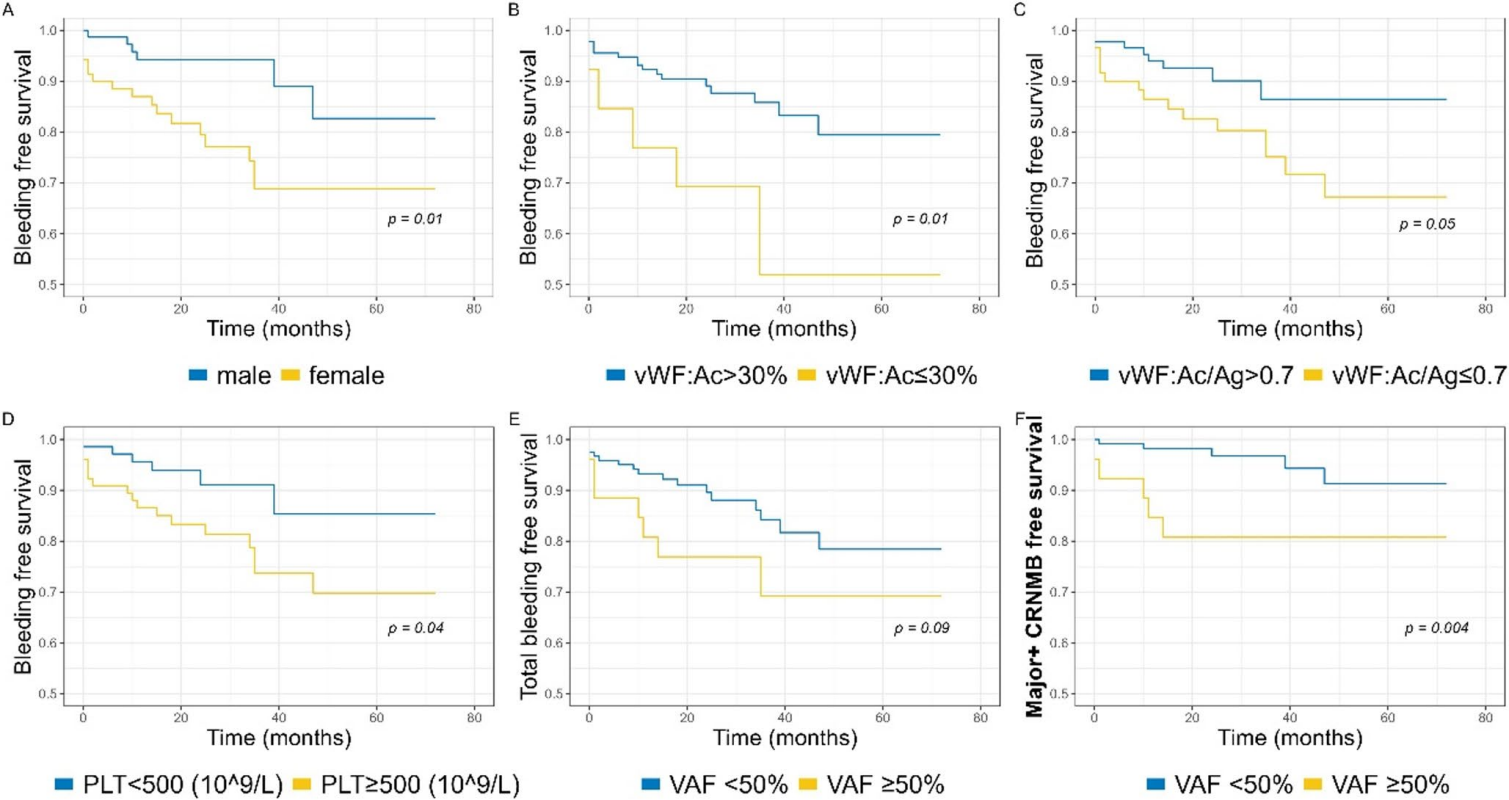
Kaplan Meier analysis of selected risk factors and their association with total (including minor) bleeding free survival (**A**-**E**) or major and clinically relevant non-major bleeding free survival (**F**). (**A**) patient sex; (**B**) vWF activity; (**C**) vWF: Activity-to-antigen ratio; (**D**) platelet count; (**E**, **F**) JAK2V617F variant allele frequency

To prospectively evaluate the risk of bleeding events conferred by clinical and laboratory parameters, we performed Kaplan Meier analyses of 149 patients without previous bleeding at the time of laboratory testing, who were followed for a median of 3.3 years. Female sex was associated with a higher risk of total bleeding events; this association could not be attributed to menorrhagia (Fig. 3A). vWF: Ac of ≤ 30%, vWF: Ac/Ag of ≤ 0.7, PLT of ≥ 500*10^9^/L and treatment with anticoagulation (*p* = 0.04) were also associated with a higher risk of total bleeding events, while extreme thrombocytosis (platelets > 1000*10^9^/L) was not (Fig. 3B-D). The presence of *JAK2*V617F and *JAK2*V617F VAF of ≥ 50% were both near-significantly associated with a higher risk of total bleeding (Fig. 3E). However, when restricting the analysis to major bleeding and CRNMB, only *JAK2*V617F VAF ≥ 50% was significantly associated with an increased risk of bleeding (Fig. 3F).

## Discussion

We evaluated the contribution of comorbidities, disease manifestations, driver mutation status and laboratory markers to thrombosis and bleeding risk assessment, in a heterogeneous and unselected MPN patient population. We observed a significant association between older age, cardiovascular comorbidities, constitutional symptoms and elevated levels of vWF: Ag, vWF: Ac and FVIII, and thrombotic events in univariate analysis. Hypertension and elevated vWF: Ag levels remained independent in multivariate analysis. Patients without previous thrombosis with vWF: Ag > 120% had a higher risk of incident thrombosis. Bleeding events were more prevalent in female and non-PV patients and were independently associated with higher *JAK2*V617F VAF. In prospective follow-up, while vWF: Ac ≤ 30%, vWF: Ac/Ag ≤ 0.7 and thrombocytosis conferred a higher risk of total (including minor) bleeding events, *JAK2*V617F VAF of ≥ 50% was the only factor predictive of major and clinically relevant non-major bleeding.

The strong association observed between vWF: Ag levels and both arterial and venous thrombosis in patients with MPN is a novel finding. To the best of our knowledge, only two studies addressed vWF levels in MPN in the context of thrombosis. In a study from 2020, high levels of vWF: Ag were observed in patients with PV as compared to both ET and healthy subjects, suggesting the use of vWF profile for assessing thrombotic risk in PV; however, clinical outcomes were not reported [24]. A recent study demonstrated an association between high levels of vWF: Ag and splanchnic vein thrombosis specifically in patients with *JAK2*V617F-positive MPNs [25]. Our findings add to these observations by confirming an association of elevated vWF levels to arterial as well as venous thrombosis and by demonstrating their predictive value.

Two studies have suggested a mechanistic role of elevated vWF levels in MPN-related thrombosis: *JAK2*V617F-positive endothelial cells from both human and mouse models led to a prothrombotic state via increased surface expression of the adhesion molecule P-selectin and vWF, as well as increased release of vWF [26, 27]. It is also possible that interindividual genetic variation in vWF, not associated with the MPN pro-inflammatory state, contributes to a pro-thrombotic profile. A meta-analysis of genome-wide associations studies reported that a 1-standard deviation increase in genetically predicted vWF levels was associated with a 2.4-fold increased risk of VTE [28, 29].

In contrast to the association observed between vWF: Ag levels and thrombosis, an association between AVWS and bleeding in patients with MPN has been previously reported [20, 21, 30, 31]. In this study, this association was replicated in prospective follow-up. Consistent with previous studies, AVWS was more prevalent in patients with ET than PV and was associated with total bleeding events which included a large proportion of minor bleeds, but not with major and clinically relevant non-major bleeding. Neither bleeding events nor AVWS were associated with extreme thrombocytosis.

Great interest has been drawn to the relevance of driver mutation VAF in MPN risk assessment in the context of thrombosis and disease progression [32–34], however, little is known about its association with bleeding. A previous prospective study observed a correlation between higher *JAK2*V617F VAF and bleeding in patients with predominantly PV and ET [35]. In this study, *JAK2*V617F VAF ≥ 50% was associated with a higher risk of major and clinically relevant non-major bleeding in a more heterogenous MPN patient population, further supporting this observation.

A limitation of this study is the prolonged interval between MPN diagnosis and laboratory testing in some of the patients, which, along with a high rate of cytoreductive treatment, may have masked the effect of variables such as blood counts and MPN risk at diagnosis. Nevertheless, cardiovascular comorbidities, vWF profile and elevated *JAK2*V617F VAF remained significant risk factors, highlighting their relevance throughout the disease course and under various treatments. Since our retrospective analysis included thrombosis events preceding laboratory testing and occasionally preceding diagnosis, and our prospective analysis was restricted to patients without thrombosis history, our analyses could not account for the effect of previous thrombosis on thrombosis risk. Strengths of this study include the documentation of both bleeding and thrombotic events, the prospective follow-up, and the inclusion of a heterogenous MPN patient population representing various disease states. Notably, the MF patients included in this study had mostly early or proliferative-phase MF, and were predominantly post-ET or -PV MF, a patient population highly relevant to thrombosis and bleeding risk assessment that is not well-represented in most studies. Lastly, the markers included in the coagulation testing are widely available in clinical practice, requiring no specialized laboratory, enhancing the applicability of our findings.

In conclusion, we found that elevated levels of vWF: Ag were associated with an increased risk of both arterial and venous thrombosis in MPN patients, AVWS was associated with an increased risk of total (including minor) bleeding, and JAK2*V617F* VAF ≥ 50% was associated with an increased risk of major and clinically relevant non-major bleeding. These findings support the utility of vWF profile and *JAK2*V617F VAF in risk stratification of both thrombosis and bleeding in MPN, either at diagnosis or later in the disease course. We suggest their use in routine evaluation in all MPN cases.

## Data Availability

Raw data and supplementary information can be provided by the corresponding author upon request.
